# Participant recruitment for paediatric research using social media: A practical ‘how‐to’ guide for researchers

**DOI:** 10.1111/1747-0080.12810

**Published:** 2023-05-08

**Authors:** Sarah Lang, Kaitlin Day, Emma Gallaher, Hiba Jebeile, Clare E. Collins, Louise A. Baur, Helen Truby

**Affiliations:** ^1^ Department of Nutrition, Dietetics and Food, School of Clinical Sciences Monash University Notting Hill Australia; ^2^ School of Agriculture and Food Faculty of Science, University of Melbourne Parkville Australia; ^3^ Opyl Ltd St Kilda Australia; ^4^ Children's Hospital Westmead Clinical School The University of Sydney Westmead Australia; ^5^ The Children's Hospital at Westmead Institute of Endocrinology and Diabetes Westmead Australia; ^6^ School of Health Sciences, College of Health, Medicine and Wellbeing University of Newcastle Newcastle Australia; ^7^ Food and Nutrition Research Program Hunter Medical Research Institute Rankin Park Australia; ^8^ Weight Management Services The Children's Hospital at Westmead Westmead Australia; ^9^ School of Human Movement and Nutrition Sciences The University of Queensland Brisbane Australia

**Keywords:** adolescent, child, communications media, patient selection, social media

## Abstract

**Aim:**

Social media platforms are being increasingly used to support participant recruitment into paediatric health‐related research. This study aimed to develop a multi‐phase approach for using social media as a recruitment strategy for paediatric research studies.

**Methods:**

The process was informed by the authors’ prior experiences recruiting for paediatric obesity‐related research studies, expertise in social media marketing and digital participant/ patient recruitment. Reflection on these experiences resulted in the iterative creation of a draft process which was further refined. A narrative literature review using a structured search was conducted to refine and augment the content and finalise the process.

**Results:**

A six‐phase recruitment approach was developed that includes: (i) plan for social media use as a recruitment strategy, (ii) explore relevant ethical considerations to protect the wellbeing of potentially vulnerable groups and create an ethical management plan, (iii) identify and understand the different target audiences and develop the advertising strategy, (iv) develop and design campaign content, (v) implement, monitor and iteratively refine the recruitment campaign, (vi) evaluate the campaign success. Potential activities and key considerations relevant for paediatric research are presented within each phase.

**Conclusion:**

Due to the widespread use and diverse characteristics of social media users, social media has the potential to disseminate details of research opportunities to community members who may otherwise not hear about, engage with, and potentially benefit from research participation. Researchers should collaborate with communication experts and target audiences to generate relevant and effective recruitment campaigns. Researchers should implement processes to protect vulnerable audiences’ wellbeing at each stage of the process. Recruitment via social media may support wider community participation in research studies designed to improve young people's health.

## 1 INTRODUCTION

A large proportion of study budgets may be directed at strategies to recruit and retain participants in clinical trials.^1^ It has been repeatedly reported that recruitment into paediatric interventions is challenging and time‐consuming.^2^, ^3^, ^4^ Social media is increasingly used as a platform to support participant recruitment into research.^5^, ^6^, ^7^, ^8^, ^9^, ^10^, ^11^, ^12^ Social media refers to online platforms through which users can create online communities to share personal messages, information and content.^13^ Social media users are diverse, with varying demographics, social backgrounds and interests.^8^ In 2021, Australian teenagers used an average of four social media services^14^ and 83% of Australian adults used social media on a daily basis.^15^ Both adults^16^ and adolescents^17^ report having accessed health‐related information via social media. Due to the widespread use and diverse characteristics of social media users, social media has the potential to disseminate details of research opportunities to community members who may otherwise not hear about, engage with, and potentially benefit from research participation.

Social media may support efficient and timely participant recruitment. A 2017 systematic review found that participant recruitment via Facebook resulted in lower costs, shorter recruitment periods, and improved participant selection in young and hard‐to‐reach demographic groups compared to traditional approaches.^5^ Implementing recruitment strategies that encourage engagement of participants from diverse backgrounds will promote an inclusive research approach and will help to build an evidence base representative of the broader population.

Participant recruitment using social media is a multi‐stage process and should involve planning, developing content, implementing and refining the campaign, and evaluating campaign success.^18^, ^19^, ^20^, ^21^ A key component of the process is identifying and developing content that resonates with the target audience.^18^, ^19^, ^20^, ^22^ In the context of paediatric research studies, audiences may include older adolescents (>16 years), parents/caregivers, and those who can promote engagement such as healthcare professionals.^23^ Tailored content for each audience can be distributed on social media platforms using paid advertisements, which involves paying for the placement of advertisements on relevant platforms, or content can be shared organically within existing online communities at no direct advertising cost to the research team.^7^

Despite the potential for social media to aid participant recruitment, health researchers often report varying results when using social media for recruitment. A recent scoping review exploring participant recruitment for medical research studies found that although 40% of the included studies identified social media as the most effective recruitment method, 17% of eligible studies recruited <5% of total participants via social media.^6^ An evidence‐based process to guide campaign development and implementation will help to streamline the process and highlight key considerations for designing effective campaigns. Existing articles provide guidance for recruitment in different populations and contexts.^18^, ^19^, ^20^, ^21^, ^24^ This article aims to develop a strategic, practical, and phased approach for participant recruitment in paediatric health‐related research using social media.

## 2 METHODS

This article presents the development of a multi‐phase approach based on literature and our prior experiences with recruiting for paediatric obesity‐related research studies and industry expertise in social media marketing^25^, ^26^, ^27^ and digital participant/patient recruitment.^28^, ^29^, ^30^, ^31^ A subset of authors met to discuss their experiences recruiting using social media. A draft process for social media recruitment was iteratively created and sent to all authors for consideration. Authors drew on their own experiences, provided comments, and the process was updated.

A narrative review of the broader literature was then completed to refine and augment the recruitment approach. An initial literature search was conducted in September 2020 using search terms relating to ‘social media’, ‘participant recruitment’, ‘research’ and ‘frameworks or guidelines’ (see Data S1). There were no limits on date of publication. The search was updated in November 2022. Overall, the intent of this article is not to systematically review the search findings, but rather to use this literature to inform a tailored, practical process. The search terms were broad to capture a range of articles providing practical guidance for social media recruitment. Recruitment processes from the general literature were reviewed and tailored to paediatric populations. The narrative review did not involve human subjects and did not require review by a Human Research Ethical Committee.

## 3 RESULTS

A six‐phase approach to participant recruitment using social media was developed. As social media is rapidly evolving with the popularity and regulations of platforms constantly changing, broad strategies that can guide the development of recruitment campaigns on different platforms were described (Table 1). The process should be iterative, and researchers may need to adjust the process to suit the specific requirements of their research project. This process can be used to support recruitment for various types of research studies, including but not limited to clinical trials, intervention and observational studies. Key terms are defined in Table 2.

**TABLE 1:** Summary of phases and key recommendations when using social media for participant recruitment for adolescent‐related research.

Phase	Key considerations and recommendations
Phase 1—Planning	Develop a clear plan outlining the project timeline, recruitment targets, staffing, budgets and resources available for online recruitment Consider if expert input is required
Phase 2—Create an ethical management plan	Develop an ethical management plan that outlines strategies to protect the wellbeing of vulnerable audiences at each phase of the recruitment process. This plan may recommend using online filters alongside outlining responsibilities and processes for staff when monitoring and responding to comments
Phase 3—Identify and understand each target audience	Identify the target audiences. Children (<16 years) are vulnerable and cannot provide informed consent. Children should never be a target audience of a social media recruitment campaign. Seek guidance from an Ethical Review Committee or Institutional Review Board if considering directly advertising to older adolescents (16–18 years) There is no ‘one size fits all’ recruitment campaign. Build an understanding of each audience's needs, values, interests, and habits. Engage the target audience to assist with campaign development where possible
Phase 4—Design and develop content	Consider the online behaviour of the target audience. Use this knowledge to select a social media platform to advertise with
	Develop advertisements and content tailored to each target audience, ideally using a co‐design approach. A consistent style, colour scheme and a study logo can help to unify the advertising approach. Select images or videos that are likely to resonate with the target audience. Check for any copyright restrictions associated with the content
	All advertising content and images should promote inclusive health messaging
	Develop a landing page with a clear call to action and a secure participant‐management process to facilitate participant enrolment in the study. Seek approval for the campaign design and content from an ethics committee
Phase 5—Implement and monitor	Consider the study timeline before commencing the online campaign. Ensure that there is adequate staffing to manage peaks in recruitment and minimise time delays in responding to potential participants
	Pilot paid advertisements before implementing the campaign. Implement paid advertisements in 3‐day blocks with a 2–4‐day break between blocks
	Monitor and iteratively adapt the campaign based on audience engagement. Assess what the audience is responding to and adjust the campaign accordingly
	Inform the ethics committee of any potential adverse outcomes of the campaign alongside any major deviations from the approved plan
Phase 6—Evaluate	Reflect on the target audience's engagement, including which audience was most successfully recruited and why. Review available metrics and use this data to inform the next campaign

**TABLE 2:** Key terms and definitions when recruiting participants via social media.^32^

Key term	Definition
Followers	The number of people who have liked or followed an account on social media
Hashtags (#)	A hashtag is used to associate a message with a common discussion or trending topic. It can also be used to casually express a mood
Impressions	The total number of times an advertisement or post has been displayed to a viewer
Influencer	A user on social media with a large audience who can drive awareness about a trend, topic, company, or product
Landing page	A single webpage or social media page that an individual is directed to after clicking on an advertisement
Meme	A picture with a humorous caption that is shared online
News feed	A regularly updated list of content posted by the social media accounts that a user follows on social media
Online group	A public or private group within a social media network in which individuals can share information about a common interest
Organic content/unpaid advertising activities	Free content such as posts, photos, videos, memes, and stories that all users, including businesses and brands, can share on their pages or news feeds
Page	An information page on social media created by organisations, businesses and public figures to share information with their customers or public
Paid advertisement	Advertisers pay a fee to direct an advertisement to a specific audience.
Post	Any social media status update, photo, video, or an item shared with other users on a blog or forum
Profile	A profile is where individuals share information about themselves such as photos or interests

***Phase 1*** involves considering and planning the recruitment approach in the early phases of the project development. Researchers should decide whether social media will be used as a recruitment strategy and consider whether timelines and staffing are adequate to support recruitment via social media.^21^, ^24^, ^33^ The total time required to develop and monitor the social media recruitment approach will depend on the extent of the campaign. Previous studies have indicated that staffing commitment can vary from 1 to 2 hours per week^19^ to having one half‐time research assistant in charge of developing and implementing the campaign.^18^

Researchers should consider available resources and develop a budget for staff, campaign development and implementation costs. The cost of a social media campaign will vary depending on the target audiences, recruitment targets, platform and advertising strategy. Darko et al.^9^ reviewed the cost‐effectiveness of recruitment via social media and traditional methods. Although they could not directly compare cost‐effectiveness between studies, they found that recruitment costs ranged from US$1.46 to US$1426 per participant when recruiting via social media compared to US$7.29 to US$2762 for other or traditional recruitment approaches.^9^

Social media platforms often operate on a pay‐per‐click model or pay for the total impressions, where advertisers pay a fee for each click or the total impressions of the advertisement or post. This fee may be higher when advertising to niche or hard‐to‐reach audiences.^32^ A recent integrative review found that the cost of implementing a campaign on Facebook ranged from US$377.69 to US$11,103.25 for the entire campaign, with recruitment targets and advertising fees impacting the overall cost.^7^

Expert support with campaign development is often recommended.^34^ Relevant industry‐based expertise can include support managing digital advertising campaigns, social media marketing, design, strategy and analytics, and specialists in website design or public relations.^18^, ^20^, ^33^, ^34^ When using social media to recruit for paediatric research, we found that engaging industry‐based experts with relevant technical expertise can help streamline campaign development and implementation. Elliot et al.^20^ reflected that engaging an expert industry media partner to support the development of a recruitment campaign resulted in compelling digital content that was executed more efficiently and effectively than traditional academic‐led dissemination campaigns. Experts can provide valuable insights on aligning the advertising approach with the platform's terms of use, along with any implications of advertising on a certain platform, including data ownership.

***Phase 2*** involves implementing strategies to protect the well‐being of vulnerable audiences at each stage of the recruitment process. Ideally, Phases 1 and 2 should be implemented concurrently. It is important to consider the vulnerability of the study population from the project's outset. Children, adolescents and families engaging with health‐related research may be vulnerable, with the extent of that vulnerability dependent on the individual, family and their circumstances. Vulnerable families may include those less likely to engage with healthcare, such as families with low incomes, young or sole parents, families from Indigenous or culturally and linguistically diverse communities, and families living with a disability or social challenges.^35^ Children (<16 years) are likely to be vulnerable and cannot provide informed consent. We recommend that researchers do not target any advertising or recruitment strategies towards children.

Researchers should develop an ethical management plan outlining staff responsibilities and processes that aim to minimise the risk of harm to vulnerable audiences during campaign development and implementation.^36^ The ethical management plan should outline strategies to mitigate the risk of negative interactions with the campaign. It is recommended that filters are set only to allow the page administrators to post on study pages. Profanity filters can be set to automatically block comments containing offensive language or keywords known to be harmful to the target population.

The ethical management plan should highlight how the research team will respond to disrespectful or malicious interactions with the campaign. This may include developing indicators that warrant a comment being hidden or developing generic responses to comments that perpetuate misconceptions or misinformation regarding the study. Stigmatising comments may be considered an adverse outcome of the campaign, especially if the comment is not quickly hidden and is viewed by vulnerable audience members. In this instance, comments should be saved in a secure folder and sent to the Principal Investigator. In some cases, comments may need to be reported to the overseeing Ethics Committee or Institutional Review Board.^36^ Vulnerable community members may be negatively affected by malicious comments in some circumstances. If the audience includes adolescents or community members who are highly vulnerable, such as individuals with eating disorders or depression, the advertisements’ landing page should contain links to appropriate support services for individuals who may be affected but are not directly engaged in the research study.

The ethical management plan should outline staff responsibilities when monitoring the campaign, including how frequently the campaign will be checked. Comments can be a valuable way for the community to engage, ask questions, and for researchers to provide feedback.^37^ We suggest checking the campaign at least once per day and recommend setting alerts to indicate when new comments are received. It is recommended that at least two staff members monitor the campaign, and responsibilities are rotated to avoid staff burnout in longer campaigns and provide greater coverage, particularly when working with large communities.^36^

Data security and safety should be considered to prevent the disclosure or loss of sensitive information collected during a research study. Identifiable participant information is unlikely to be stored securely on a social media platform. Researchers should familiarise themselves with local guidelines and recommendations for safe data storage and integrate key principles into their ethical management plan.^38^

***Phase 3*** involves identifying the target audiences, being the key groups or stakeholders that the research team aims to recruit or connect with on social media.^18^, ^19^, ^20^, ^22^ Researchers then develop a deeper understanding of the interests and online behaviours of each target audience.

The research team will need to identify the target audiences of the recruitment campaign. There will likely be multiple target audiences. Within the context of paediatric research studies, target audiences may include parents/caregivers, older adolescents, or study advocates, such as primary care physicians or school nurses. Each audience will likely consist of multiple micro‐audiences defined by common geographic or demographic traits alongside similar preferences, media habits, lifestyle habits, and values.^39^ Recognising and tailoring the campaign to each micro‐audience will support the development of tailored content more likely to appeal to the individuals the research team seeks to engage.

Parents/caregivers are likely a key target audience, with this audience consisting of multiple micro‐audiences. For example, the advertising approach for parents of primary school‐aged children will differ from an advertising approach for parents of adolescents.

Children are often not permitted to create personal social media accounts,^40^ are highly vulnerable, and cannot provide informed consent. We believe that children (<16 years) should never be a target audience of a social media recruitment campaign.

Older adolescents (16–18 years) may be a suitable audience for the advertising campaign. However, adolescents can be vulnerable to online advertising. Social media platforms often have strict policies when advertising to adolescents, especially content relating to weight loss, tobacco, alcohol, and gambling.^40^ Although Facebook has previously been used to directly recruit adolescents, a recent systematic review reported that researchers implemented additional processes to ensure informed assent or consent was obtained appropriately from both adolescents and parents/guardians.^41^ Additional precautions to avoid disseminating potentially harmful messages to this vulnerable population are essential. We recommend seeking guidance from an Ethical Review Committee or Institutional Review Board if considering advertising to older adolescents.

Finally, study advocates, such as health professionals, teachers, school principals or staff, sports club coaches and community leaders, may be a potential audience for a recruitment campaign. These advocates can disseminate study details, which can create confidence in the study among potential participants.^23^ Advocates may refer multiple participants to the study, potentially resulting in a greater return for the total investment. However, researchers need to ensure that no undue influence or pressure has been placed on families to participate by advocates. Appropriate processes for attaining informed consent and assent should be in place to mitigate this risk (in Phase 2).

The research team should build an understanding of the differing interests, social media preferences, and online behaviours of each micro‐audience to support the development of tailored campaign content for each audience. Researchers will need to consider the condition being investigated and tailor their advertising strategy accordingly. Individuals will be more likely to engage with an advertisement if it is personally meaningful or relevant.^42^ For example, the advertising approach to engage older adolescents will differ from the approach used to engage parents/caregivers. Understanding the preferences of each audience will support the development of tailored content that resonates with each audience's specific psycho‐demographics, online behaviour, and beliefs.^43^

Co‐design workshops are a useful strategy to build an understanding of each audience and support the development of tailored advertisements. Co‐design involves actively engaging key stakeholders, including community members and representative members of the target audience, such as parents, and researchers, to decide on the advertising approach and design advertisements collectively.^44^ Decisions are iteratively made in a group or workshop setting.^44^ Elliott et al.^20^ reported that social media knowledge translation strategies had an extended reach and higher level of engagement when co‐designed by patient or parent stakeholders compared with advertisements created solely by researchers. Engaging or consulting with community members from under‐represented populations or families on the feasibility and acceptability of the virtual recruitment approach may be useful to support the development of tailored content.^45^

If co‐design workshops are not possible, we suggest researchers reflect upon prior experiences and refer to the literature exploring each target audience's interests and online behaviours. We then suggest developing personas or hypothetical representations of audience members to characterise the target audience's needs. From our experiences, personas or adopting participants’ viewpoints can support researchers to synthesise common motivations or preferences within each audience and envisage the target audience's perspectives.^46^

Researchers should familiarise themselves with the options, opportunities, and resources available for advertising and recruitment across the different social media platforms. An expert on social media can often provide valuable insights into the pros and cons of using each platform for recruitment.^20^

The target audience's online behaviour and preferences should determine which platform/s is the most appropriate to advertise on. Social media is rapidly evolving, with platforms’ popularity, usage and functions constantly changing. A 2022 integrative review by Darko et al.^9^ identified that researchers report using Facebook, Twitter, Craigslist, Instagram, YouTube, LinkedIn, Reddit, Snapchat, and Tumblr, in addition to blogs and WhatsApp Messenger for recruitment.

Understanding the target audience and platform will enable the research team to select the most appropriate platform to engage each audience. At the time of writing this review, Twitter is considered useful in reaching members of the health research or healthcare community,^47^ whereas researchers seeking to reach adolescents may consider YouTube, Instagram, or Snapchat.^48^ TikTok is popular among preadolescents, so we suggest being cautious to avoid inadvertently advertising to children if advertising on this platform.^49^, ^50^ The demographics of Facebook users are diverse, with individuals of varying ages, contexts and geographical locations active on the platform. Facebook is, therefore, a popular platform for participant recruitment.^7^ Facebook may not be suitable to reach older adolescents as they are currently less likely to engage with this platform.^48^ Individuals of certain ages, ethnicity, country and socioeconomic status may more readily engage with a specific platform.

We suggest initially selecting only one or two platforms for recruitment. Building an understanding of each platform's regulations, functionalities, and policies regarding suitable advertising approaches may be time‐intensive. Communications or social media marketing experts familiar with the different platforms’ functionalities and requirements can help streamline this process.^20^

The research team will need to develop an advertising strategy, which considers whether paid advertisements, unpaid advertising activities, or complementary recruitment strategies will be used. Paid advertising involves paying a fee to direct an advertisement towards a target audience. In contrast, unpaid advertising activities involve sharing information with existing communities with no direct advertising costs to the research team.^7^ Filters for disseminating paid advertisements to target audiences can be highly specific. For example, Facebook advertisers can filter audiences based on age, gender, location, demographics, online behaviour, and specific or niche interests.^51^ A clear understanding of the target audience will support the appropriate use of these filters.

An unpaid advertising approach often involves sharing content with private or public groups. Researchers must seek permission before sharing advertisements with online groups or community pages. Rattani et al.^52^ recommend that researchers engage a community gatekeeper, being a leader within the online community, to be an intermediary between researchers and online communities. This will minimise researcher interference within the group, help prevent coercion, protect community members, and avoid interfering with the dynamics of the online community, especially if it is being used as a support network.

Engaging a leader or expert with a pre‐existing online presence, specialised knowledge, authority or insight among the target audience may be a useful complementary advertising strategy.^53^ Credible community leaders or healthcare professionals can create trust in the research while disseminating the details of the project.^20^, ^54^ Elliott et al.^20^ observed that collaborating with a parent who understood the nature of the research and the target community created interconnectedness and increased the perceived credibility of the campaign. We often engage healthcare professionals to share the details of a research project with their existing online networks.^29^, ^30^, ^31^, ^55^, ^56^

Having a study social media page for the research project can help create an online presence and increase the visibility of the study. Larger studies may benefit from setting up a social media account specifically for the research study. These pages may be useful to post unpaid advertisements and can be used as a landing page for paid advertisements^57^ or disseminate study findings to participants. Developing an online community on social media may be a useful long‐term strategy to aid recruitment for similar research projects in future. However, building an online community is often resource‐intensive.^57^, ^58^ The research team will likely need to use strategies, such as paid advertisements or hashtags, to support the target audiences to find the page. Researchers are then encouraged to regularly upload relevant content, such as links to evidence‐based information, blogs, or helpful suggestions to promote and maintain community engagement.^58^, ^59^ Strategies that require an action, such as ‘sign ups’ or polls and tagging, can prompt audience engagement and help maintain their interest.^59^ Building an online community are likely outside the scope of small or short‐term projects. In these circumstances, researchers may choose to connect the study with the social media pages of research organisations or institutions overseeing the study, as the brand and reputation of a trusted institution can influence the perceived trustworthiness of the advertisement.^60^, ^61^

***Phase 4*** involves developing campaign content and advertisements tailored to each target audience. Content should be developed with a clear and concise message, a relevant image or video, consistent branding, and an eye‐catching design. Macapagal et al.^18^ propose that advertisements follow a NICE heuristic, being noticeable, intriguing, credible and engaging. The message should succinctly and clearly convey the relevance of the research study to the reader.^62^ Language should be tailored to align with the target audience's preferences and norms of each platform, as this can increase the perceived credibility of the message.^60^ Previous research in paediatric populations suggests parents engage with positive language that shows enthusiasm.^23^ In contrast, adolescents have indicated a preference for positive, encouraging, and direct messages that avoid colloquial language.^63^ Understanding each micro‐audience will highlight their preferences regarding appropriate terminology, enabling researchers to tailor language according.

When selecting an image or video, previous research has indicated that adults prefer images they can connect with emotionally, such as family or group photos.^22^ Alternatively, Kearns et al.^64^ developed advertisements with an animated comic and found that it was very effective in recruiting often‐difficult to recruit Maori adults. A short video explaining your project in lay terms may also effectively communicate your project.^65^

A consistent design and colour scheme across the different advertising approaches can help to create a cohesive study identity.^23^ A study brand, being a name, design or feature that identifies this research project as unique from others, and a study logo can unify the advertising approach and increase credibility.^39^, ^66^ We suggest developing a suite of advertisements with differing features to pilot and inform which copy, call to action, image, style or post has the best engagement with the target audience.^18^, ^19^, ^20^

All advertising content should be ethically responsible, use inclusive people‐first language, and avoid alienating, blaming an individual or exacerbating stereotypical views. Weight bias and stigma are highly pervasive on online platforms. Posts on social media can blame individuals for their health status or perpetuate ideas of inadequacy, negatively impacting health outcomes.^67^ Health professionals need to promote inclusive health messaging and use positive, non‐stigmatising images in all online communications. Content messages should also avoid linking a health condition to an individual.^68^ For example, the phrase ‘does your child have depression?’ allows families to inadvertently disclose their child's health status, whereas ‘study to support parents of children with depression’ does not prompt a disclosure.

Consider the current regulations of the social media platform and copyright restrictions when selecting images, videos and designs. Ensure appropriate consent has been obtained if using images of people.^69^

The research team will need to develop a landing page, being a page on a social media platform or webpage that an individual is directed to after clicking on the advertisement.^70^ Landing pages should briefly convey the study details to the reader and are not necessarily the study website homepage. The landing page should include a call‐to‐action, being a device designed to prompt a response from the audience.^70^ The call‐to‐action may include assessing eligibility or completing an online expression of interest form. The audience should be able to easily navigate to pages with additional information.

We suggest developing a landing page tailored to each of the target audiences. For example, viewers clicking on an advertisement for health professionals are directed to a page with relevant information for health professionals. When recruiting using social media, Akers and Gordon^19^ initially developed a research‐oriented landing page with a text‐heavy description. As audience engagement was poor, the page was revised to include friendlier language, additional graphics and user testimonials. These revisions increased the average number of participants enrolling after clicking on the advertisement from 0.5 to 3 participants per day, indicating that the design of the landing page can be equally as important as the design of the advertisements.

The research team will need to consider the participant management process or process from when an individual views the advertisement to expressing interest and enrolling in the study.^71^ A website with an online expression of interest form is likely to be most effective in supporting enrolment. Requesting participants to switch to another medium, such as phone or email, is potentially burdensome and may negatively impact enrolment.^19^ Any confidential data provided by potential participants must be secure. It is unlikely that social media platforms will support a secure and confidential expression of interest or enrolment process. An existing electronic data capture program may be useful to manage the enrolment process. Researchers may benefit from expert advice on a suitable participant management process.^19^

The research team will need to consider strategies to deter fake or fraudulent enrolments if the study is primarily online.^72^ Pozzar et al.^72^ reflected that studies recruiting via social media are susceptible to data quality issues. Opportunistic individuals may use virtual private servers to fraudulently complete research surveys for profit. They suggest implementing strategies within the study protocol and Institutional Review Board ethical application, using data collection tools with fraud protection, and actively monitoring post and data collection for fraudulent engagement.

Researchers should also implement processes to ensure that participants understand the requirements of the study before enrolling. Macapagal et al.^18^ recommend using a mix of automated and staff‐initiated tasks, such as eligibility screeners, comprehension checks and identification verification. Monitor, streamline and rapidly respond to participants to ensure that this process does not become a barrier to recruitment.^18^

The research team will need approval from a human research ethics committee or Institutional Review Board for the campaign strategy and content. These committees will likely require a detailed overview of the campaign strategy, including an outline of target audiences, key messages, and images that may be used during the campaign, and a risk assessment.^73^ We recommend highlighting to the committee that although the campaign's overarching message will not change, each advertisement may be iteratively refined to align with platform guidelines or more effectively meet the target audiences’ needs after piloting the advertisements.

***Phase 5*** involves implementing, monitoring, and iteratively refining the advertising campaign. If social media is used alongside other recruitment strategies, it may be beneficial to implement all advertising approaches concurrently. Anecdotal reports suggest that parents often need to hear about a study on several occasions and from several sources before making a formal enquiry.^4^

The process of uploading or creating advertisements will differ between social media platforms. Once uploaded, the advertisement will be subjected to a review process to ensure it meets platform regulations. Researchers may need to submit multiple iterations of the advertisement, with changes to wording, messages or images, before the advertisement is approved.

If using paid advertisements, we suggest initially piloting advertisements to understand what style of advertisement is most effective in engaging the audience.^18^ This involves disseminating advertisements with different combinations of the copy, call to action, image, style or post.^18^, ^19^, ^20^ We suggest piloting advertisements for 4–7 days on the selected platform/s. Review available metrics (Table 3) to determine which style of advertisement is most effective in engaging the audience and remove poor‐performing advertisements from the campaign. Review the audiences’ comments, questions, and assess for any common misconceptions. Use these comments and misconceptions to refine the wording and key messages on advertisements for clear communication.

**TABLE 3:** Example metrics to monitor the campaign success adapted from key performance indicators by Neiger et al.^74^

Measure	Metric	Definition
Exposure, that is, the number of times content is viewed	Impressions	Total number of times an advertisement or post has been displayed to a viewer
	Reach	Total number of unique accounts that have seen the advertisement or post
	Total views	Total views of a video
Engagement, that is, the number of people who have acknowledged or interacted with content	Actions on page	Total clicks on contact information and the call‐to‐action
	Audience characteristics	Demographics of audience engagement with the advertisement
	Audience growth rate	The rate that the number of fans, followers or friends are increasing
	Audience ratings	Total page likes
	Click‐through rate	Ratio of users who click on a specific link to the number of total users who view the advertisement or post
	Conversion rate	Ratio of visits via social media to the webpage to enrolment in the study
	Cost‐per‐click	Fee charged when an audience member clicks on the advertisement or link
	Cost‐per‐impression	The advertising cost for each view or impression of an online advertisement or post (usually calculated per 1000 impressions)
	Engagement rate	Total likes, comments, reviews or shares
	Recommendations	Total number of people who recommended the page. This may include account mentions, retweets and shares
	Total followers	Number of people who follow or subscribe to the page

We suggest disseminating paid advertisements in 3‐day blocks with a 2–4‐day break between blocks following piloting. These short blocks support repeat exposures to advertisements and maximise the use of available resources by extending the campaign timeline without the cost of running the campaign full‐time. Avoid advertising on the same 3 days each week and include both weekdays and weekends. If using unpaid advertisements, consider the best time to share posts. For example, posting in the afternoon or evening may increase the visibility of the post.^75^

The campaign requires close and continual monitoring while live to review interactions with the campaign, implement the ethical management plan, and document relevant metrics.^20^ The implementation process should be iterative. Researchers may need to update platform filters, re‐evaluate spending on advertisements, or adjust advertisements based on audience engagement. Engagement with advertisements will likely decrease over time. The decision to stop advertising may depend on the budget or pre‐determined timelines. Akers and Gordon^19^ recommend waiting for engagement to remain relatively dormant for 2 days before ending the advertising campaign. If the engagement levels are dwindling yet the recruitment target has not been reached, it may be necessary to refresh the campaign by changing the content, swapping to an alternative platform^18^ or changing the platform's filters to direct the advertisement to audiences that are more difficult to recruit. Researchers may choose to return to Phase 2 of the process to better understand the target audience's motivations and refine the campaign accordingly.

***Phase 6*** involves evaluating the campaign's success in facilitating participant recruitment within the required timeframe. We suggest collecting data on where participants initially heard about the study to evaluate the effectiveness of different recruitment approaches in engaging the target population.

Researchers can review the overall campaign impact and reach by analysing platform metrics,^74^, ^76^ assessing the total resources invested in the campaign, reviewing demographics of participants who heard about the study via social media compared to traditional recruitment approaches, and parent/caregiver and adolescent satisfaction with the enrolment process.^22^

User comments on social media can be qualitatively analysed to assess audience engagement.^76^ However, in most circumstances, the user has not provided consent for the comment to be used for research purposes. Researchers should only collect data from pages or advertisements that are widely understood by the general audience to be public, and research teams are transparent that they may collect data from these pages.^77^ Privacy disclaimers can be added to pages or advertisements to clearly indicate that posts or comments are publicly available.^68^ Finally, Macapagal et al.^18^ recommended saving any negative comments as they can be used to inform the ethical management plans for future campaigns.

## 4 DISCUSSION

This article offers a practical and comprehensive guide for recruitment into paediatric research studies using social media (summarised in Table 4). The process is based on the existing literature alongside our prior experiences recruiting to nutrition and weight management research programs in Australia and industry expertise. This process can be iteratively adapted to meet the research team's and study participants’ needs. We acknowledge that social media is rapidly evolving, and guidelines and regulations of platforms are regularly updated. As these platforms evolve, the process for recruitment using social media may change. Processes may differ between countries, and there may be additional considerations when recruiting in other settings. This article described a practical process derived from the author's experiences. As such, we do not describe quantifiable evidence on the outcomes of using this process in practice, which is a limitation of this narrative review. Finally, to ensure research findings represent the broader population, social media may need to be used in conjunction with other recruitment approaches to facilitate the recruitment of individuals who do not use social media.

**TABLE 4:** Checklist for researchers when recruiting using social media.

**Recruitment Checklist** ***Planning*** ☐ Create a recruitment plan and budget ☐ Engage a social media expert ***Ethical considerations*** ☐ Create an ethical management plan outlining staff responsibilities, management of negative interactions with the campaign, and data safety. ***Design your social media strategy*** ☐ Identify your target audience ☐ Engage the target audience to co‐design the advertising strategy and content ☐ Select a social media platform to advertise on ☐ Determine an advertising strategy for each platform ☐ Develop a social media presence for the study ***Design and develop content*** ☐ Create ethical content/ advertisements (consider the message, images, branding, design, and call to action) ☐ Develop the landing page ☐ Develop a secure participant management process ☐ Seek ethical approval for campaign content ***Implement and monitor*** ☐ Pilot advertisements ☐ Implement and monitor campaign performance ***Evaluate*** ☐ Use metrics to evaluate campaign success

Overall, this article describes a strategic, iterative multi‐phased social media recruitment approach that can be adapted to meet the needs of each research project. Effectively harnessing social media's reach may help enhance the dissemination of the research studies to the wider community. Social media may support traditionally hard‐to‐reach groups, children, adolescents, and families to access treatments and interventions that may provide more health professional support than would otherwise be available via public health services.

## AUTHORS CONTRIBUTIONS

Substantial contributions to conception and design, acquisition of data, or analysis and interpretation of data [SL, KD, EG]. Drafting the article or revising it critically for important intellectual content [SL, KD, EG, HJ, CEC and LAB, HT]. Final approval of the version to be published [SL, KD, EG, HJ, CEC and LAB, HT].

## FUNDING INFORMATION

Ms. Sarah Lang is supported by an Australian Government Research Training Program (RTP) Scholarship. Dr Hiba Jebeile is supported by the Sydney Medical School Foundation (The University of Sydney). The funder/sponsor had no role in the design and conduct of the study. CEC is supported by a National Health and Medical Research Council of Australia Leadership (L3) Research Fellowship (APP2009340).

## CONFLICT OF INTEREST STATEMENT

Emma Gallaher is employed by Opyl Ltd, an organisation that provides social media and online clinical trial recruitment services. This work was conducted independently from their employer and reflects their experiences as an industry expert in this area. Helen Truby is an Editorial Board Member of *Nutrition & Dietetics*. They were excluded from the peer review process and all decision‐making regarding this article. This manuscript has been managed throughout the review process by the Journal's Editor‐in‐Chief. The Journal operates a blinded peer review process and the peer reviewers for this manuscript were unaware of the authors of the manuscript. This process prevents authors who also hold an editorial role to influence the editorial decisions made. All other authors have no competing interests to declare.

## Supporting information


**Data S1:** Supporting Information

## Data Availability

Data sharing is not applicable to this article as no new data were created or analysed in this study.
