# Supplementary material for: Participant recruitment for paediatric research using social media: A practical ‘how‐to’ guide for researchers
Source: Nutr Diet. 2023 May 8;80(4):338–50. doi: 10.1111/1747-0080.12810 (PMC10952907; doi:10.1111/1747-0080.12810)
Supplement: Supplementary file 1 — Data S1: Supporting Information [file NDI-80-338-s001.docx]

**Participant recruitment for pediatric research using social media: A guide for researchers**

# **Supplementary File**

# **Search Strategy**

## ***Database Searches***

**DATABASE:** MEDLINE(R) and Epub Ahead of Print, In-Process & Other Non-Indexed Citations, Daily and Versions(R)

**Host:** Ovid

**Strategy:**

1. “social media”. ti,ab.
2. Exp social Media/
3. ((patient* or participant*) adj3 recruit*).ti,ab.
4. (Research* or trial* or program*). ti,ab.
5. (Process* OR guide* OR model* OR framework* OR review). ti,ab.
6. 1 OR 2
7. 3 AND 4 AND 5 AND 6

**DATABASE:** EMBASE

**Host:** Ovid

**Strategy:**

1. “social media”. ti,ab.
2. Exp social Media/
3. ((patient* or participant*) adj3 recruit*). ti,ab.
4. (Research* or trial* or program*).ti,ab.
5. (Process* OR guide* OR model* OR framework* OR review) .ti,ab.
6. 1 OR 2
7. 3 AND 4 AND 5 AND 6

**DATABASE:** SCOPUS

**Host:** Elsevier

**Strategy:**

1. TITLE-ABS-KEY (“Social Media”)
2. TITLE-ABS-KEY ((Patient* OR participant*) W/3 recruit*)
3. TITLE-ABS-KEY (Process* OR guide* OR model* OR framework* OR review)
4. TITLE-ABS-KEY (Research* or trial* or program*)
5. TITLE-ABS-KEY (1 AND 2 AND 3 AND 4)

**DATABASE:** Web of Science Core Collection

**Host:** Clarivate

**Strategy:**

(AB=(“Social Media” ) AND AB=((Patient* Near/3 recruit*) OR (Participant* Near/3 recruit*)) AND AB=(Process* OR guide* OR model* OR framework* OR review) AND AB=(Research* or trial* or program*)) OR (TI=(“Social Media” ) AND TI=((Patient* Near/3 recruit*) OR (Participant* Near/3 recruit*)) AND TI=(Process* OR guide* OR model* OR framework* OR review) AND TI=(Research* or trial* or program*))

**DATABASE:** ProQuest Central

**Host:** Cambridge Information Group

**Strategy:**

1. (abstract("Social Media") AND abstract((Patient* OR participant*) NEAR/3 recruit*) AND abstract(Process* OR guide* OR model* OR framework* OR review) AND abstract(Research* OR trial* OR program*)) OR (title("Social Media") AND title((Patient* OR participant*) NEAR/3 recruit*) AND title(Process* OR guide* OR model* OR framework* OR review) AND title(Research* OR trial* OR program*))
2. Limit to scholarly journals
